# Supplementary material for: Complexome profiling of the Chlamydomonas psb28 mutant reveals TEF5 as an early PSII assembly factor
Source: Plant Cell. 2025 Mar 17;37(6):koaf055. doi: 10.1093/plcell/koaf055 (PMC12164590; doi:10.1093/plcell/koaf055)
Supplement: koaf055_Supplementary_Data [file koaf055_supplementary_data.zip › psb28_interactive.zip!/plots/intensity/qualityPlots/peptides/NUOA1_Cre10.g459750.t1.2.html]

### Cre10.g459750.t1.2

Trivial name: NUOA1  
SeqCoverage [median]: 23.10  
Mass: 7.64 kDa  
  
 

| Sequence | MissedCleavage | Mass [Da] | wtIntensity | muIntensity | Score |
| --- | --- | --- | --- | --- | --- |
| DWYLQREEETLAMAK | 1 | 1881.89 | 4651.28 | 1944.38 | 69.72 |
| EEETLAMAK | 0 | 1020.48 | 688.41 | 618.43 | 74.25 |
|  |  |  | 5339.7 | 2562.8 |  |
